# COVID-19 frontline primary health care professionals’ perspectives on health system preparedness and response to the pandemic in the Mahalapye Health District, Botswana

**DOI:** 10.4102/phcfm.v14i1.3166

**Published:** 2022-04-19

**Authors:** Stephane T. Tshitenge, Justus M. Nthitu

**Affiliations:** 1 Department of Family Medicine and Public Health, Faculty of Medicine, University of Botswana, Gaborone, Botswana; 2 Department of Occupational Health, Mahalapye District Hospital, Botswana Ministry of Health and Wellness, Mahalapye, Botswana

**Keywords:** COVID-19, health system preparedness and response, primary health care professionals, Botswana

## Abstract

**Background:**

The World Health Organization issued interim guidelines on essential health system preparedness and response measures for the coronavirus disease 2019 (COVID-19) pandemic. The control of the pandemic requires healthcare system preparedness and response.

**Aim:**

This study aimed to evaluate frontline COVID-19 primary health care professionals’ (PHC-Ps) views on health system preparedness and response to the pandemic in the Mahalapye Health District (MHD).

**Setting:**

In March 2020, the Botswana Ministry of Health directed health districts to educate their health professionals about COVID-19. One hundred and seventy frontline PHC-Ps were trained in MHD; they evaluated the health system’s preparedness and response.

**Methods:**

This was a cross-sectional study that involved a self-administered questionnaire using the Integrated Disease Surveillance and Health System response guidelines.

**Results:**

The majority (72.5%) of participants felt unprepared to deal with the COVID-19 pandemic at their level. Most of the participants (70.7%) acknowledged that the health system response plan has been followed. About half of the participants attributed a low score regarding the health system’s preparedness (44.4%), its response (50.0%), and its overall performance (55.6%) to the COVID-19 pandemic. There was an association between participants’ age and work experience and their overall perceptions of preparedness and response (*p* = 0.009 and *p* = 0.005, respectively).

**Conclusion:**

More than half of the participants gave a low score to the MHD regarding the health system’s preparedness and response to the COVID-19 pandemic. Further studies are required to determine the causes of such attitudes and to be better prepared to respond effectively.

## Introduction

Coronavirus disease 2019 (COVID-19) – a disease caused by the virus called severe acute respiratory syndrome coronavirus 2 (SARS-CoV-2) – has spread worldwide with more than 180 million confirmed cases and about 4 million deaths because of COVID-19 from late December 2019 to July 2021.^1^

The World Health Organization (WHO) African region (AFRO) introduced the Integrated Disease Surveillance and Health System Response (IDSR) strategy in 1998, with the goal of efficiently integrating different surveillance and health system responses^1^; the IDSR guidelines provide a checklist to measure the health system’s preparation and the team’s reaction to priority illnesses, conditions and occurrences.^2^ Some African nations integrated lessons learned from diseases such as the Ebola virus disease into the COVID-19 pandemic health system response.^3^ With the recent pandemic, the WHO issued interim guidelines on essential health system preparedness and response measures for COVID-19, with the goal of controlling COVID-19, slowing viral transmission and preventing related disease and death.^4^

Preparedness involves actions prior to a pandemic; it includes planning and coordination meetings, standard operating procedures, training staff and ensuring that necessary equipment is available. Whilst response pertains to actions taken after a pandemic has occurred, it aids in mitigating negative consequences.^5^ Preparedness pertains to the hardware and software of a health system. The WHO, which summarised core domains of health systems in a six-building-block framework, condenses the health system in two systems, ‘hardware’ and ‘software’.^6^ Health system ‘hardware’ – focused on material resources and structures – includes surveillance, infrastructure and medical supplies, workforce and communication mechanisms. Whilst health system ‘software’ – human and institutional relationships, values and norms – includes governance and trust.^7^ Palagyi A et al. reported that the substance of health system preparedness provides health professionals with the required knowledge and information, as well as keeps them up to date on developing situations, actions and choices as they occur.^7^ During the planning phase, it is essential to ensure that information is distributed evenly amongst primary healthcare professionals (PHC-Ps) and other stakeholders.^7^

Wolfe et al. reported that 47 articles about IDSR were published between 2012 and 2019 in 17 African countries and regional level systems.^8^ Only four articles focused on assessing health workers perspectives^9,10,11,12^; three articles of the studies involved multiple diseases,^9,10,11^ whilst one focused on a single disease – avian influenza.^12^ Although IDSR was first implemented in Botswana in 2001, only one study examining the utilisation of the new malaria case-based surveillance system has been published from the country thus far.^13^ Studies that evaluate PHC-Ps’ views on COVID-19 health system preparedness and response used the IDSR framework which is scarce.

The present study used the IDSR criteria to assess frontline COVID-19 PHC-Ps’ perspectives on health system readiness and reaction to the COVID-19 pandemic in the Mahalapye Health District (MHD).

## Methods

### Study setting, design and period

This was a cross-sectional study using a self-administered questionnaire in the MHD from 20 April 2020 to 08 May 2020.

The MHD is a rural health district in central Botswana, some 200 km from the country’s capital city, Gaborone. It contains 46 healthcare facilities.

The Botswana Ministry of Health and Wellness (MoHW) instructed all health districts to educate their health professionals about COVID-19 in March 2020, shortly following the quarantine of the first suspected COVID-19 case in Botswana. In the MHD, a COVID-19 Preparedness and Rapid Health System Response Team (COVID-19 PRHSRT) of 20 health professionals (doctors, nurses and allied health workers) was constituted. From March to mid-April 2020, in a series of 1-day workshops (6 h each), 170 frontline COVID-19 PHC-Ps (in groups of 30–40 participants) were trained on COVID-19 management in the area and demonstration of the use of personal protective equipment (PPE). The COVID-19 PRHSRT allocated frontline COVID-19 PHC-Ps to local clinic teams with specific tasks. Also, MoHW, with technical assistance from the WHO Country Office, conducted an IDSR training in November 2020 to strengthen public health surveillance and response systems to COVID-19, targeting district health management teams (DHMT) in Botswana’s southern region, including Gaborone.^14^

### Study population and recruitment

At the end of the COVID-19 management training, the Mahalapye Quality Management Team introduced the research topic to all heads of departments and staff involved in frontline management of COVID-19. Frontline COVID-19 MHD PHC-Ps who received COVID-19 management training from the COVID-19 PRHSRT between March and mid-April 2020 (*n* = 170) were purposively sampled for the study. This included medical doctors, nurses, allied health professionals, pharmacists, radiology and laboratory scientists who work in frontline management of COVID-19.

A self-administered questionnaire was then distributed to all 170 PHC-Ps. Participants were asked to drop the questionnaire in a designated box after completing it at the end of the day.

### Instrument, data collection and analysis

A questionnaire based on the IDSR guideline served as the data collection tool.^2^ The questionnaire encompassed three sections: (1) the demographic information (participants’ work unit, gender, age group, qualification, profession and work experience); (2) a 19-item section to appraise the health system’s preparedness (governance and trust, surveillance, workforce, infrastructure and medical supplies and communication mechanisms-community engagement)^2,7^ and (3) a 16-item section to assess health system response such as the COVID-19 PRHSRT committee in your local area/health facility, clarity of roles and responsibilities of members and a pandemic health system response plan being followed in the health facility.^2^

Participants were instructed to respond with ‘yes’ if they agreed with the item’s statement, ‘no’ if they disagreed and ‘don’t know’ if they weren’t sure or weren’t aware.

A ‘yes’ answer received one point, whilst a ‘no’ or ‘don’t know’ answer received no point. The total number of points for each component of the questionnaire was then tallied. The level of health system preparedness was judged poor when the score was 0–6, moderate (7–12) and high (13–19). When the score was 0–5, a low level of health system response was considered, a moderate level when the score was 6–11 and a high level when the score was 12–16. The overall performance of the MHD’s health system preparedness and response to COVID-19, as reported by participants, was calculated by adding the preparedness and response scores, yielding a total score of 35. Low performance was assigned a score of 0–12, moderate performance was assigned a score of 13–25 and excellent performance was assigned a score of 26–35.

The data distribution was summarised by calculating the mean ± standard deviation (s.d.) for normally distributed variables, and the frequency in percentages for binomial and the median ± interquartile range (IQR), if skewed. A Chi-squared test was performed to assess the association between dependent variables (level of health system preparedness, level of health system response and the overall performance of COVID-19 in MHD) with regard to independent demographic variables. All statistical analyses were carried out using R software version 3.0.0 with an R commander package version 1.9–6.^15^ The level of significance was set at *p <* 0.05.

## Results

Of the 170 questionnaire forms that were issued, 151 (89%) forms were returned. Nine (2.7%) forms had incomplete demographic information, therefore they were discarded. For analysis, we considered 142 questionnaire forms. Statements were fully answered in 132–141 cases, but only 116 frontline COVID-19 PHC-Ps responded to the statement about infrastructure and medical supplies being ready.

Table 1 illustrates the demographic profile of participants. Eighty-eight (58.3%) participants were female.

**TABLE 1:** Demographic profile of frontline healthcare professionals who participated in the health system preparedness and response to COVID-19 survey, Mahalapye District Health, April 2020 – May 2020 (*N* = 142).

Demographic information	Male (*n* = 54) (38%)	Female (*n* = 88) (62%)	Total	
			*N*	%
**Age group (years)**				
20–29	11	18	29	20.4
30–39	25	39	64	45.1
40–49	13	20	33	23.2
50–59	4	10	14	9.9
≥ 60	1	1	2	1.4
**Profession**				
Allied	9	16	27	17.6
Medical officer	11	8	19	13.4
Nurse	30	61	91	64.1
Pharmacist	4	3	7	4.9
**Work experience (years)**				
0–3	8	12	20	14.1
4–7	9	17	26	18.3
8–11	8	20	28	19.7
12–15	12	11	23	16.2
16–19	7	11	18	12.7
≥ 20	10	17	27	19.0

About one in two (45.1%) participants were from the 30- to 39-year age group and two-thirds (64.1%) of the participants were nurses.

Table 2 summarises MHD frontline PHC-Ps’ views on the health system’s preparedness for the COVID-19 pandemic.

**TABLE 2:** Health system preparedness for the COVID-19 pandemic: Views of frontline primary health care professionals, Mahalapye District Health Management Team, April 2020 – May 2020 (*n* = 142).

Core constructs of emerging infectious disease	Questions about health system preparedness for the COVID-19 pandemic in MHD	Yes		No		Don’t know		Total respondents
		*n*	%	*n*	%	*n*	%	
Governance and trust	Are you aware of a COVID-19 PRHSRT in MHD?	87	63.0	16	11.6	35	25.4	138
	Are the COVID-19 PRHSRT meets frequently?	37	26.4	18	12.9	85	60.7	140
	Are there established roles and responsibilities for committee members?	46	33.6	19	13.9	72	52.6	137
	Are there sub-committees to assign to specific tasks?	36	26.3	25	18.2	76	55.5	137
	What are ethical issues anticipated and planned for?	35	25.7	36	26.5	65	47.8	136
Surveillance	Are there established plans for tackling disease outbreak?	62	46.3	20	14.9	52	38.8	134
	Is there a protocol for the investigation of an outbreak?	60	45.1	21	15.8	52	39.1	133
	Do you have surveillance systems in place?	43	30.9	44	31.7	52	37.4	139
	Is the surveillance system effective?	17	12.7	43	32.1	74	55.2	134
	Is there an established referral system?	98	71.0	14	10.1	26	18.8	138
Workforce	Are healthcare staff trained in health system preparedness and response?	90	67.2	30	22.4	14	10.4	134
	Do you feel prepared in dealing with the pandemic at your level?	32	23.2	100	72.5	6	4.3	138
	Are there staffing adjustments made to improve the numbers?	54	38.8	65	46.8	20	14.4	139
	Is there availability of volunteers and peripheral health staff?	21	15.2	68	49.3	49	35.5	138
Infrastructure and medical supplies	Is there availability of stock for essential supplies?	37	28.0	57	43.2	38	28.8	132
	Is there a shelter for site isolation?	105	77.8	20	14.8	10	7.4	135
	There is an established local, regional and national laboratory for confirmation of COVID-19 test results.	87	75.0	16	13.8	13	11.2	116
Communication mechanisms-community engagement	Is there provision for educating the community on disease outbreaks?	93	66.4	16	11.4	31	22.1	140
	Has community education on COVID-19 been sufficient?	53	37.6	49	34.8	39	27.7	141

COVID-19, coronavirus disease 2019; COVID-19 PRHSRT, COVID-19 Preparedness and Rapid Health System Response Team; MHD, Mahalapye Health District.

Eighty-seven (63.0%) participants were aware of the existence of a COVID-19 PRHSRT in MHD; however, they did not know whether meetings were held frequently (60.7%) or whether COVID-19 PRHSRT members had established roles and specific responsibilities within the committee (55.5%). Ninety-eight (71.0%) participants acknowledged that there was an established referral system for COVID-19 patients in MHD, but more than half (55.2%) of the participants did not know whether the surveillance system was effective. The majority (72.5%) of participants felt unprepared to deal with the COVID-19 pandemic at their level. Two-thirds (67.2%) of the participants reported that they were aware of the existence of a shelter for site isolation, whilst 100 (72.5%) admitted that there was an established local, regional and national laboratory for confirmation of COVID-19 test results. Ninety-three participants (66.4%) agreed that there was a provision for educating the community on disease outbreaks.

Table 3 summarises MHD frontline PHC-Ps’ views of the health system’s response to the pandemic. Although half of the participants (48.1%) did not know about a local/ health facility COVID-19 PRHSRT committee, half of the participants recognised that their committee meets at least weekly during the pandemic period (57.4%), with the committee composed of administration personnel (57.4%). However, less than a third of participants (27.9%) reported that there was a clarity of roles and responsibilities of members of the COVID-19 PRHSRT local/health facility committee. The majority (70.7%) of participants acknowledged that the pandemic health system response plan has been followed in the health facility, but denied that PPE were adequately available in MHD (70.4%). Twenty-eight percent (39/139) of participants felt that there was an attempt to limit spread and take precaution in protecting the staff, whilst only 28.7% (39/136) of participants reported that the protocol for investigation of COVID-19 cases was implemented. Ninety-six (69.6%) of participants reported that supply of food and other personal items for the isolated cases had been available from the onset.

**TABLE 3:** Health system response for the COVID-19 pandemic: Views of frontline primary health care professionals, Mahalapye District Health Management Team, April 2020 – May 2020 (*N* = 142).

Statements about health system response for the COVID-19 pandemic in MHD	Yes		No		Don’t know		Total respondents
	*n*	%	*n*	%	*n*	%	
There is a COVID-19 PRHSRT committee in your local area/health facility	38	27.1	34	24.3	68	48.6	140
The COVID-19 PRHSRT local/health facility committee meets at least weekly during the pandemic period	72	51.4	38	27.1	30	21.4	140
The COVID-19 PRHSRT local/health facility committee inclusive of admin personnel	81	57.4	39	27.7	21	14.9	141
There is clarity of roles and responsibilities of members	38	27.9	43	31.6	55	40.4	136
The team is mandated to handle all pertinent issues on COVID-19	29	21.2	58	42.3	50	36.5	137
Is the pandemic health system response plan being followed in the health facility?	94	70.7	14	10.5	25	18.8	133
There was further training and use of volunteers to help with screening patients	23	16.8	24	17.5	90	65.7	137
PPE was available and adequate	15	11.1	95	70.4	25	18.5	135
There was an attempt to limit the spread and take precaution in protecting the staff	39	28.1	53	38.1	47	33.8	139
There is check of movement in and out of the health facility(s)	45	32.8	28	20.4	64	46.7	137
Protocol for investigation was implemented	39	28.7	18	13.2	79	58.1	136
Cases/suspected cases were quarantined/isolated	46	34.3	18	13.4	70	52.2	134
Supply of food and other personal items for the isolated cases was available from the onset	96	69.6	6	4.3	36	26.1	138
Surveillance data are being developed and are used to monitor the control of the disease	54	39.1	8	5.8	76	55.1	138
Community awareness about COVID-19 was raised within a week	28	20.4	16	11.7	93	67.9	137
There is an established data reporting/communication system	37	26.6	7	5.0	95	68.3	139

COVID-19, coronavirus disease 2019; COVID-19 PRHSRT, COVID-19 Preparedness and Rapid Health System Response Team; PPE, personal protective equipment; MHD, Mahalapye Health District.

About half of the participants credited a low score regarding the MHD health system preparedness to the COVID-19 pandemic (44.4%), its response (50.0%) and overall performance (55.6%) (Table 4). There was no statistical difference between the participants’ credited scores regarding their demographic profiles – age and performance on COVID-19 (*p* = 0.10), work experience and health system response to COVID-19 (*p* = 0.52). However, there was a statistical difference between the overall performance (in preparedness) to the COVID-19 pandemic with regard to age (*p* = 0.009) and work experience (*p* = 0.005) with older and more experienced professionals.

**TABLE 4:** Overall performance of health system preparedness and response to the COVID-19 pandemic: Views of primary health care professionals, Mahalapye District Health Management Team, April 2020 – May 2020 (*N* = 142).

Demographic information	HS preparedness to COVID-19 score			*p*	HS response to COVID-19 score			*p*	Overall performance			*p*
	Low	Mod.	High		Low	Mod.	High		Low	Mod.	High	
**Age-groups**	-	-	-	0.10	-	-	-	0.90	-	-	-	0.009
20–29	16	10	3	-	16	12	1	-	18	10	1	-
30–39	30	25	9	-	38	23	3	-	40	23	1	-
40–49	14	9	10	-	12	16	5	-	16	11	6	-
50–59	3	6	5	-	5	9	-	-	5	9	-	-
> 60	-	2	-	-	-	2	-	-	-	2	-	-
**Work experience**	-	-	-	0.52	-	-	-	0.32	-	-	-	0.005
0–3	15	5	2	-	13	8	1	-	18	3	1	-
4–7	11	10	4	-	12	13	-	-	12	13	-	-
8–11	10	15	6	-	14	13	4	-	11	19	1	-
12–15	10	6	4	-	14	6	-	-	16	2	2	-
16–19	7	6	4	-	6	10	1	-	8	8	1	-
> 20	10	10	7	-	12	12	3	-	14	10	3	-
**Profession**	-	-	-	0.48	-	-	-	0.22	-	-	-	0.18
Allied	9	10	5	-	15	8	1	-	13	9	2	-
MO	10	8	1	-	8	10	1	-	11	7	1	-
Nurse	42	32	18	-	45	42	5	-	53	36	3	-
Pharm.	2	2	3	-	3	2	2	-	2	3	2	-
**Total**												
*n*	63	52	27	-	71	62	9	-	79	55	8	-
%	44.4	36.6	19	-	50	43.7	6.3	-	55.6	38.7	5.6	-

*n*, number; COVID-19, coronavirus disease 2019; Mod., moderate; Pharm., pharmacists, MO, medical officers; HS, health system.

## Discussion

This study surveyed MHD frontline PHC-Ps’ views on the health system’s preparedness, response and overall performance against the COVID-19 pandemic. Although most participants (60.7%) were aware of the existence of a surveillance system and a COVID-19 preparedness and response committee in the district, around half of the participants rated preparedness, response and overall performance as low.

In MHD, the frontline COVID-19 PHC-Ps were young. In Pakistan and Libya, both developing countries like Botswana, Zafar N et al. and Elhadi et al. reported a similar frontline COVID-19 age-group with a mean age of 27 years (in Pakistan) and 35.5 ± 7.3 years for doctors and 27.8 ± 5.4 years for nurses (in Libya).^16,17^ However, frontline COVID-19 health professionals are older in high-income countries than in low-income countries, with studies from the United States and Norway, reporting a mean age of 44 years.^18,19^ With the COVID-19 pandemic, many developed countries are concerned about the ageing workforce in general, and in the healthcare sector, in particular.^20^ This could be explained by the population pyramid difference amongst low-, middle- and high-income countries. There could be an advantage of having young frontline human resources in developing countries as the COVID-19 pandemic is more likely to negatively impact older people.^21^

Although two-thirds of participants were aware of the existence of a COVID-19 PRHSRT in MHD, they knew little about its organisational and operational structure, as they did not know how frequently the committee met, whether sub-committees were assigned to specific tasks or whether members had established roles and specific responsibilities within the committee. Almost three-quarters of participants recognised the existence of a COVID-19 referral system, but more than half of them did not know whether the surveillance system was effective. This was likely why most participants felt unprepared to deal with the COVID-19 pandemic at their level. Good communication encourages collaboration and enhances the response to a pandemic.^22^ About half of the participants attributed a low score regarding the health system preparedness of the MHD, its response and the overall performance to the COVID-19 pandemic. To accomplish health system preparedness for any infectious disease outbreak, one should consider the uniqueness of software components of the health system that hold the hardware together.^7^ Knowledge of a condition is always associated with confidence in handling that condition. There was an association between participants’ age and work experience and their overall perceptions of preparedness and response (*p* = 0.009 and 0.005, respectively). Inexperienced health workers are more likely to be dissatisfied with their jobs than experienced health workers.^23^ However, when assessed individually, there was no relationship between participants’ age and work experience and their perceptions of preparedness and response (*p* = 0.10 and *p* = 0.52, respectively).

This study period, which coincided with the start of the pandemic in most African countries, could have influenced participants’ negative reactions to COVID-19 health system preparedness and response in the district, as there were many unanswered questions about the pandemic worldwide, which could have led to fear of the disease. A pilot study was not conducted to assess the questionnaire’s user-friendliness; the exercise would have most likely improved the tool’s reliability. Also, because it was not part of the study, we did not conduct post-hoc tests to determine where statistic differences in participants’ age groups and work experience lied in terms of overall perceptions of preparedness and response.

A year prior to the COVID-19 pandemic in Africa, most African countries embarked on activities to detect disparities in their capacity to prevent and respond to public health menaces – known as Joint External Evaluation (JEE) of the International Health Regulations (IHR).^24^ Joint External Evaluation recommended African countries to build up a multisectoral National Action Plan for Health Security (NAPHS) when dealing with the detected public health menaces and lining up with diverse sectoral plans. Although from these exercises, African countries gained capabilities in real-time surveillance and immunisation, the resilience of the health system to respond to an outbreak needs optimisation.^24^ We recommend that Botswana escalates the JEE recommendation to the district level for a better response to a pandemic. We also recommend that when planning training during outbreaks, learning objectives and outcomes should be customised to frontline health professionals at district level – rather than only national level learning objectives and outcomes – which addresses high levels of Bloom’s taxonomy knowledge, attitudes and behaviour of learners.^25^ A feeling of insecurity due to the perception of lacking skills to manage themselves and their clients during outbreaks could lead to more crises, faulty risk perceptions and more contamination in the community. Risk perception is a crucial aspect of life and seeing dangers as either low or too high can more likely to negatively impact a person’s well-being.^26^ For example, people who see risks as unreasonably low may expose themselves to needless hazards or risky circumstances.^26^

## Conclusion

This study surveyed MHD frontline PHC-Ps’ views on the health system’s preparedness, response and overall performance against the COVID-19 pandemic. Most participants felt unprepared to deal with the COVID-19 pandemic at their level. About half of the participants attributed a low score regarding the health system preparedness of the MHD, its response and the overall performance to the COVID-19 pandemic. We recommend that Botswana escalates the JEE recommendation to the district level for a localised response to a pandemic. Further studies are required to find out the causes of such attitudes so that they can be well prepared to respond effectively.
